# Varioloid A, a new indolyl-6,10b-dihydro-5a*H*-[1]benzofuro[2,3-*b*]indole derivative from the marine alga-derived endophytic fungus *Paecilomyces variotii* EN-291

**DOI:** 10.3762/bjoc.12.188

**Published:** 2016-09-09

**Authors:** Peng Zhang, Xiao-Ming Li, Xin-Xin Mao, Attila Mándi, Tibor Kurtán, Bin-Gui Wang

**Affiliations:** 1 Laboratory of Marine Biology and Biotechnology, Qingdao National Laboratory for Marine Science and Technology, Key Laboratory of Experimental Marine Biology, Institute of Oceanology, Chinese Academy of Sciences, Nanhai Road 7, Qingdao 266071, China, Fax: +86 532 82880645; 2 Tobacco Research Institute of Chinese Academy of Agricultural Sciences, Qingdao 266101, China; 3 Department of Organic Chemistry, University of Debrecen, P. O. Box 400, 4002 Debrecen, Hungary, Fax: +36 52 512-744

**Keywords:** bisindolyl benzenoid derivatives, cytotoxicity, marine alga-derived fungus, *Paecilomyces variotii*, TDDFT-ECD calculation

## Abstract

A new indolyl-6,10b-dihydro-5a*H*-[1]benzofuro[2,3-*b*]indole derivative, varioloid A (**1**), was isolated from the marine alga-derived endophytic fungus *Paecilomyces variotii* EN-291. Its structure was elucidated on the basis of extensive analysis of 1D and 2D NMR data and the absolute configuration was determined by time-dependent density functional theory-electronic circular dichroism (TDDFT-ECD) calculations. A similar compound, whose planar structure was previously described but the relative and absolute configurations and ^13^C NMR data were not reported, was also identified and was tentatively named as varioloid B (**2**). Both compounds **1** and **2** exhibited cytotoxicity against A549, HCT116, and HepG2 cell lines, with IC_50_ values ranging from 2.6 to 8.2 µg/mL.

## Introduction

The filamentous fungus *Paecilomyces variotii* is a ubiquitous species commonly occurring in air, compost, infected humans, and various foodstuffs [1]. This fungus is well-known for its biotechnological applications and for its ability to produce enzymes and proteins [2]. Its metabolic potential enables it to be a prolific source of bioactive secondary metabolites of diverse structures, including, for example, semiviriditoxin derivatives with antibacterial activity [3], cornexistin and hydroxycornexistin with herbicidal activity [4], and paecilocins with antibacterial activity [5].

**Figure 1:**
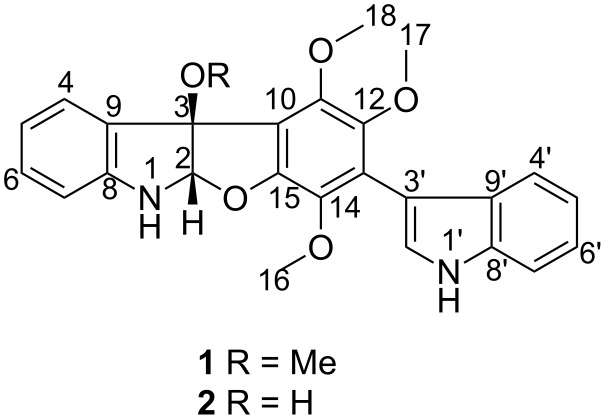
Structures of the isolated compounds **1** and **2**.

During our ongoing effort to search for structurally unique and bioactive secondary metabolites from marine fungi, especially from marine alga-derived fungi [6–8], we discovered evident DPPH (1,1-diphenyl-2-picrylhydrazyl) radical scavenging activity and diverse antimicrobial activities in the EtOAc extract from *Paecilomyces variotii* EN-291, an endophytic fungus isolated from the red alga *Grateloupia turuturu*. The investigation of the chemical constituents of this fungal strain had been performed and as a result, three new oxepine-containing alkaloids with antimicrobial activities [9–10], two new butenolide derivatives with DPPH radical scavenging activity [11], and two new prenylated indole alkaloids with cytotoxic activity [12] had been isolated and identified. In an effort to isolate additional analogues that might show similar effects, a larger fermentation was undertaken. This study led to the isolation of two cyclized bisindolyl benzenoid derivatives (compounds **1** and **2**) (Figure 1). The rare planar structure of compound **2** was previously reported but the full NMR data were not disclosed and the relative and absolute configuration had not been determined [13]. Herein we describe the isolation, structural elucidation including the assignment of the absolute configuration, and the cytotoxicity studies of these compounds.

## Results and Discussion

Varioloid A (**1**), obtained as a light brown solid, has the molecular formula C_26_H_24_N_2_O_5_ as established from a prominent pseudomolecular ion peak at *m/z* 445.1766 [M + H]^+^ in its HRESI mass spectrum, implying 16 degrees of unsaturation. The ^1^H NMR spectrum showed signals (Table 1) attributed to nine olefinic or aromatic protons (δ_H_ 6.70–7.78), four methoxy groups (δ_H_ 3.53, H_3_-16; 3.38, H_3_-17; 4.10, H_3_-18; 3.35, 3-OMe), one broad indolic NH singlet (δ_H_ 8.36, 1’-NH), and one isolated sp^3^-methine proton (δ_H_ 6.42, H-2), which were unambiguously designated by the HSQC experiment.

**Table 1:** ^1^H and ^13^C NMR data of compounds **1** and **2** (at 500 MHz for ^1^H and 125 MHz for ^13^C, measured in CDCl_3_).

Position	**1**		Position	**2**	
	
	δ_C_	δ_H_ (*J* in Hz)		δ_C_	δ_H_ (*J* in Hz)
			
2	100.3, CH	6.42, s	2	104.9, CH	6.22, s
3	96.1, C	–	3	90.2, C	–
3-OMe	52.1, CH_3_	3.35, s	3-OH	–	4.98, s
4	126.0, CH	7.78, d (7.3)	4	124.8, CH	7.72, d (7.5)
5	119.8, CH	6.90, t (7.3)	5	119.7, CH	6.83, t (7.5)
6	129.8, CH	7.19, t (7.3)	6	130.0, CH	7.14, dd (7.5, 7.9)
7	109.5, CH	6.70, d (7.3)	7	109.8, CH	6.66, d (7.9)
8	149.1, C	–	8	148.6, C	–
9	127.2, C	–	9	129.7, C	–
10	118.9, C	–	10	120.9	–
11	146.0, C	–	11	145.4, C	–
12	145.7, C	–	12	145.4, C	–
13	124.6, C	–	13	124.4, C	–
14	138.4, C	–	14	138.3, C	–
15	148.6, C	–	15	148.1, C	–
16	60.3, CH_3_	3.53, s	16	60.2, CH_3_	3.48, s
17	60.5, CH_3_	3.38, s	17	60.5, CH_3_	3.31, s
18	61.0, CH_3_	4.10, s	18	61.3, CH_3_	4.09, s
1’-NH	–	8.36, br s	1’-NH	–	8.29, br s
2’	124.5, CH	7.32, d (2.2)	2’	124.4, CH	7.26, br s
3’	108.6, C	–	3’	108.6, C	–
4’	121.1, CH	7.55, d (7.8)	4’	121.1, CH	7.48, d (8.0)
5’	119.6, CH	7.12, t (7.8)	5’	119.7, CH	7.05, t (8.0)
6’	121.8, CH	7.20, t (7.8)	6’	121.8, CH	7.16, t (8.0)
7’	110.8, CH	7.41, t (7.8)	7’	110.8, CH	7.36, d (8.0)
8’	135.8, C	–	8’	135.8, C	–
9’	126.0, C	–	9’	127.2, C	–

**Figure 2:**
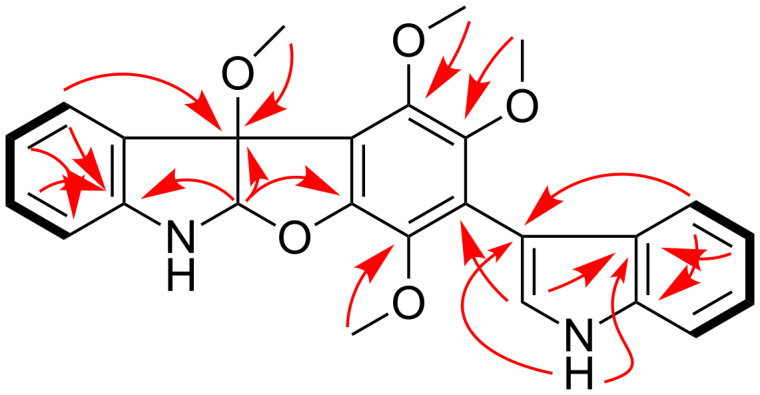
Key HMBC (arrows) and COSY (bond lines) correlations of compound **1**.

A comprehensive analysis of the COSY spectrum (Figure 2) revealed the existence of two 1,2-disubstituted benzenoid rings (sequential COSY correlations from H-4 to H-7 and from H-4’ to H-7’). HMBC correlations from the hetero-substituted methine H-2 to C-3, C-8, and C-9, from H-4 to C-3 and C-8, and from the 3-OMe to C-3 were instrumental in construction of the 3-methoxyindoline moiety, while additional HMBCs from 1’-NH to C-3’ and C-9’, from H-2’ to C-13, and from H-4’ to C-3’ enabled extension of the partial structure to 3-indolyl group. These units accounted for six quaternary aromatic carbons and three methoxy groups, requiring the presence of a hexasubstituted benzene ring bearing three methoxy groups and the two indolyl subunits as substituents. Each methoxy signal showed an HMBC correlation to its corresponding oxygenated aromatic carbon. HMBC correlation from H-2 to C-15 indicated the presence of an ether bond between C-2 and C-15. Thus, the planar structure of compound **1** was elucidated as shown in Figure 1.

The relative configuration of **1** was elucidated by the NOESY spectrum. Clear NOE correlation between H-2 and 3-OMe led to recognition that these protons adopt *cis* orientation. In order to elucidate the absolute configuration of **1,** solution TDDFT-ECD protocol [14–15] was carried out on the arbitrarily chosen (2*R*,3*R*) enantiomer.

**Figure 3:**
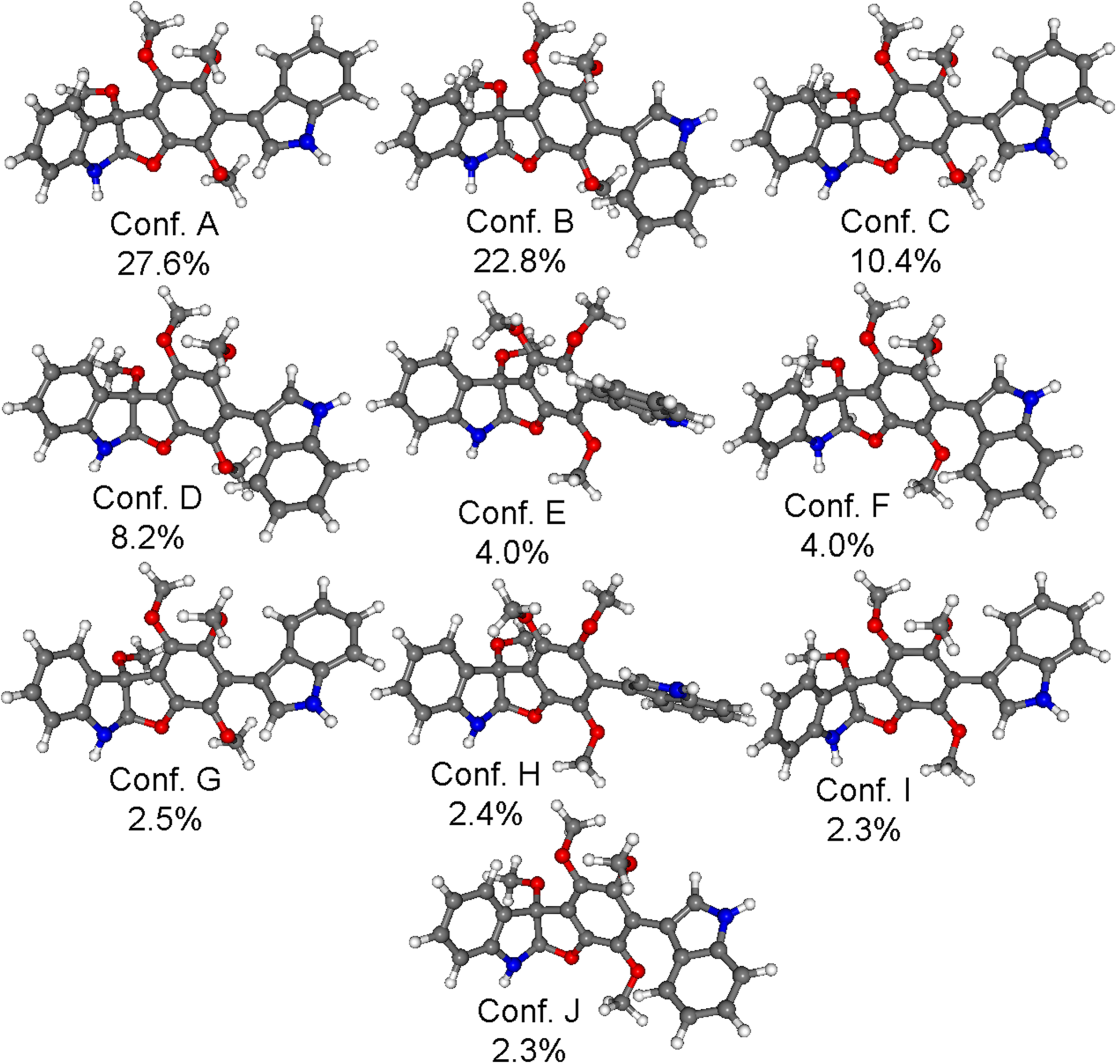
Structure and population of the low-energy B3LYP/6-31G(d) conformers (>2%) of (2*R*,3*R*)-**1**.

The preliminary conformational search at MMFF (Merck Molecular Force Field) level resulted in 55 conformers within a 21 kJ/mol energy window. These conformers were reoptimized at two different DFT levels, namely B3LYP/6-31G(d) in vacuo and B97D/TZVP [16–17] with a Polarizable Continuum Model (PCM) for MeCN [18]. The B3LYP optimization yielded 10 low-energy conformers above 2% Boltzmann population, while the number of low-energy conformers was 14 at the applied B97D level (Figure 3).

**Figure 4:**
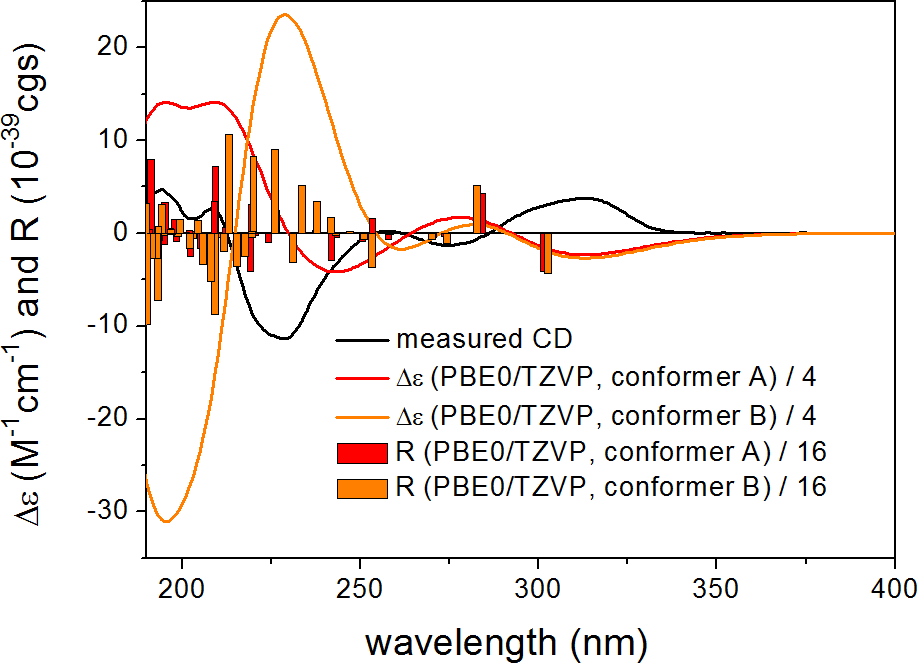
Experimental ECD spectrum of **1** in MeCN compared with the computed PBE0/TZVP spectra of the lowest-energy *M* (ω_C12−C13−C3’−C9’_ = –46.1°, conformer A) and *P* (ω_C12−C13−C3’−C9’_ = 133.4°, conformer B) helicity B3LYP/6-31G(d) conformers of (2*R*,3*R*)-**1**. Bars represent the rotational strength values of the two conformers.

The conformers differed in the orientation of the methoxy groups and the value of the ω_C12−C13−C3’−C9’_ biaryl torsional angle resulting in different orientations of the C-13 indol and dihydro-5a*H*-[1]benzofuro [2,3-*b*]indole moieties. The different biaryl torsional angles produced markedly different computed ECD spectra for the conformers having *M* and *P* helicity such as the two lowest-energy computed B3LYP/6-31G(d) conformers (Figure 4). The two high-wavelength Cotton effects (CEs) had the same sign for conformer A and B, while the lower-wavelength (<250 nm) CEs were significantly different. The hindered rotation along the biaryl linkage may have implied an additional stereogenic element, which would have enabled atropodiastereomers with axial chirality.

**Figure 5:**
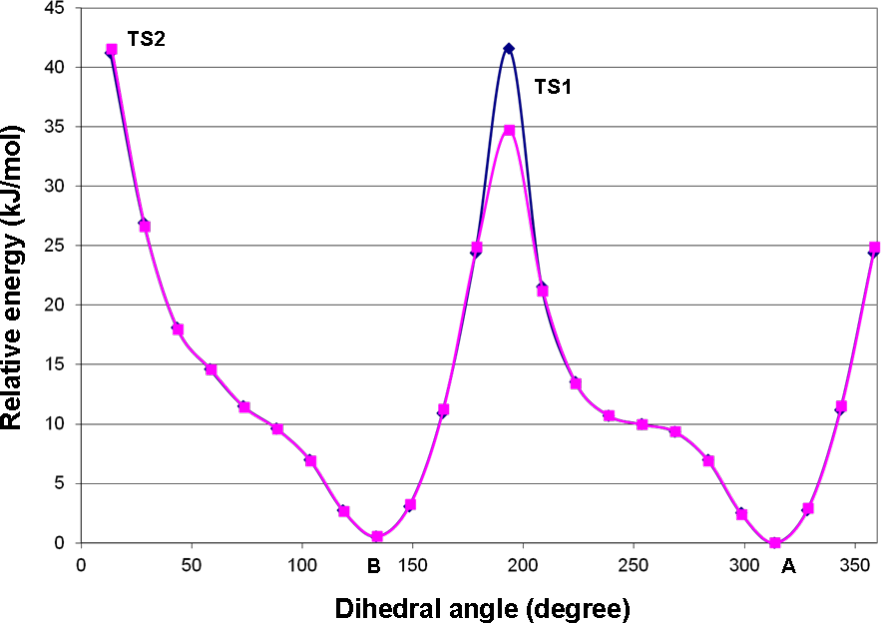
Torsional angle scans for estimating the rotational energy barrier around the C13−C3’ bond (ω_C12−C13−C3’−C9’_ torsional angle) of (2*R*,3*R*)-**1**. The scans were started from the lowest-energy in vacuo conformers with *M* and *P* helicity (conformer A and B, respectively). The relative energy (kJ/mol) is plotted as the function of the ω_C12−C13−C3’−C9_ torsional angle. TS1 and TS2 denote the two transition states for the inversion of the helicity.

**Figure 6:**
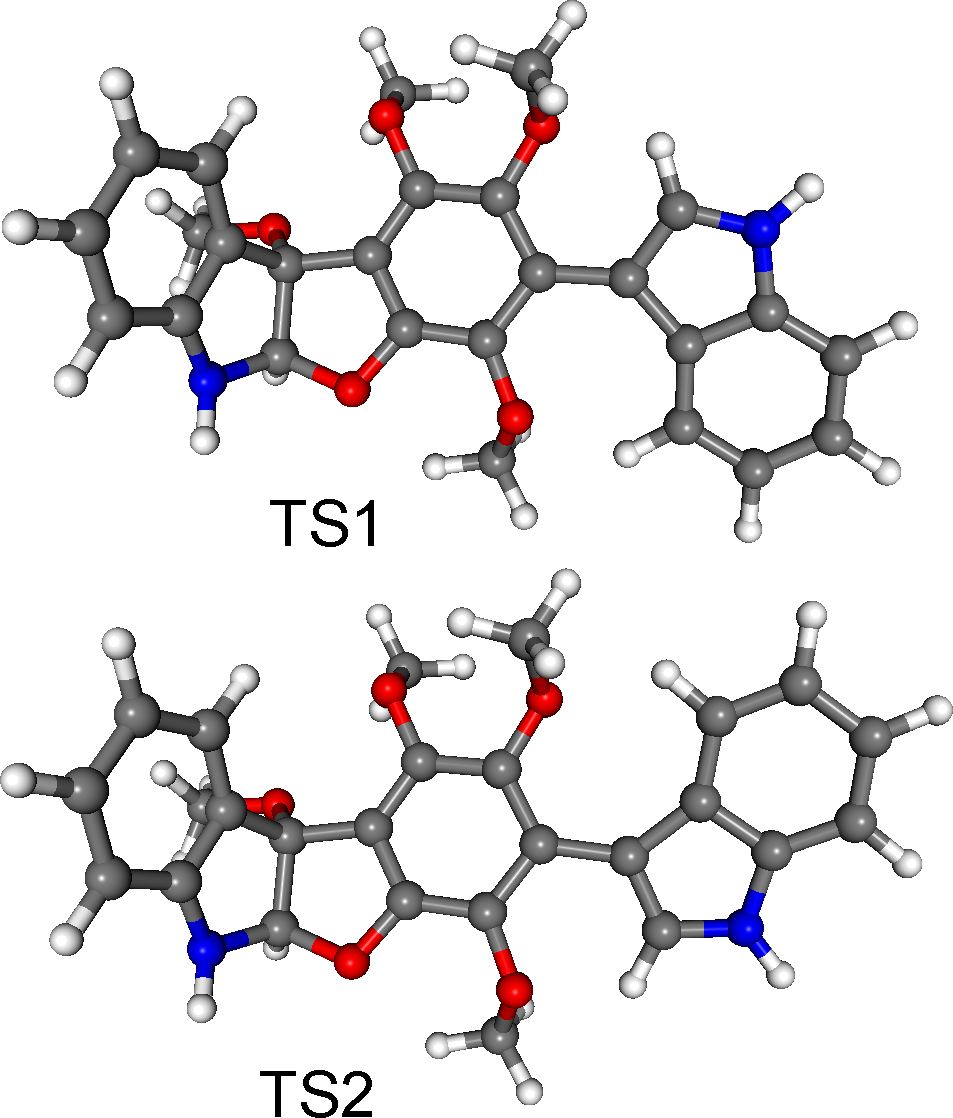
Transition states for the inversion of the helicity [TS1 (ω_C12−C13−C3’−C9’_ = 168.1°) and TS2 (ω_C12−C13−C3’−C9’_ = 14.8°)].

In order to explore the possibility of axial chirality, torsional angle scans were performed on the lowest-energy *M* (conformer A) and *P* (conformer B) helicity gas-phase conformers or atropodiastereomers. Rotational energy barriers between the two isomers were estimated to be ca. 35–42 kJ/mol for TS1 (ω_C12−C13−C3’−C9’_ ≈ 180°) and 42 kJ/mol for TS2 (ω_C12−C13−C3’−C9’_ ≈ 0°) from the preliminary torsional scans, indicating free rotation at room temperature (Figure 5). Transition state (TS) calculations started from the energy scans’ maxima resulted in TS structures with somewhat higher energies than those estimated previously [TS1 = 45.3 (46.7 with ZPVE correction) kJ/mol and TS2 = 46.5 (48.1 with ZPVE correction) kJ/mol], which were however not large enough (< ca. 93 kJ/mol) to afford hindered rotation (Figure 6) at room temperature [19].

Thus the determination of the rotational energy barriers clearly showed that there is no axial chirality in **1**, and equilibrating conformers with *M* and *P* helicity are present in solution as obtained in the conformational analysis.

**Figure 7:**
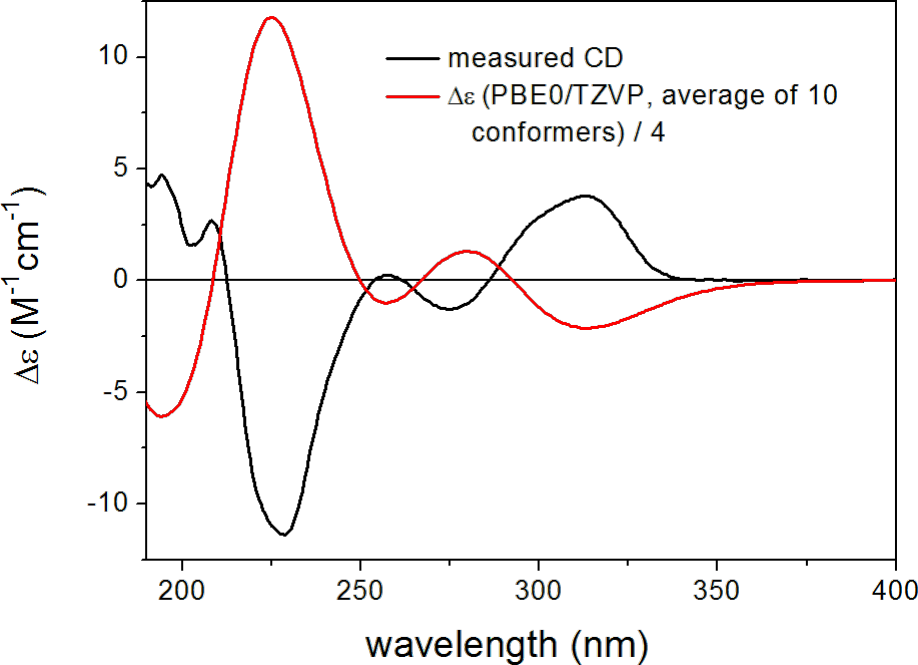
Experimental ECD spectrum of **1** in MeCN compared with the Boltzmann-weighted PBE0/TZVP ECD spectrum of (2*R*,3*R*)-**1** computed for the B3LYP/6-31G(d) conformers.

The Boltzmann-weighted ECD spectra calculated for both the gas-phase and the solvent model conformers of (2*R*,3*R*)-**1** at B3LYP/TZVP, BH&HLYP/TZVP and PBE0/TZVP levels showed good mirror image agreement with the experimental spectrum (Figure 7). The two high-wavelength CEs above 250 nm were quite independent from the value of the biaryl torsional angle, while the CEs below 250 nm showed large differences in sign and shape with the different torsional angles. The good mirror image agreement of the experimental and computed ECD curves allowed the unambiguous assignment of the absolute configuration of **1** as (2*S*,3*S*), as well as the estimation of the ratio of the *P* and *M* helicity conformers in solution.

Compound **2** was also obtained as a light brown solid and it was identified as the 3-hydroxy derivative of **1**, the planar structure of which was already reported [13]. However, the relative and absolute configurations and the full NMR data were not disclosed. The fully assigned NMR data of compound **2** was listed in Table 1 and this compound was tentatively named as varioloid B. The NOESY spectrum indicated *cis* relative configuration of the two adjacent chirality centers, which was in agreement with that of **1**. Given the common biosynthetic origin and congruent ECD spectrum with that of compound **1**, the absolute configuration of **2** was assigned to be the same as that of **1**.

Both compounds **1** and **2** were evaluated for their cytotoxic activity using a panel of three tumor cell lines, A549 (human lung adenocarcinoma cells), HCT116 (human colon carcinoma cells), and HepG2 (human hepatoma cells). Both compounds exhibited relevant cytotoxicity. Compound **1** showed potent cytotoxicity against A549, HCT116, and HepG2 cell lines, with IC_50_ values of 3.5, 6.4, and 2.5 µg/mL, respectively, while compound **2** also showed considerable activities, with IC_50_ values of 4.6, 8.2, and 6.6 µg/mL, respectively.

## Conclusion

The filamentous fungus *Paecilomyces variotii* continues to be a prolific source of bioactive secondary metabolites with diverse structures. In this paper, two indolyl-6,10b-dihydro-5a*H*-[1]benzofuro[2,3-*b*]indole derivatives, varioloids A (**1**) and B (**2**), were isolated from the marine alga-derived endophytic fungus *Paecilomyces variotii* EN-291. The condensed heterocyclic system in compounds **1** and **2** is quite rare among natural products. The absolute configuration of **1** was confirmed to be (2*S*,3*S*) by conformational analysis and TDDFT-ECD calculations. In the cytotoxicity assay, both compounds exhibited remarkable cytotoxicity. Compound **1** showed potent cytotoxicity against A549, HCT116, and HepG2 cell lines, with IC_50_ values of 3.5, 6.4, and 2.5 µg/mL, respectively, while compound **2** also showed considerable activities, with IC_50_ values of 4.6, 8.2, and 6.6 µg/mL, respectively.

## Experimental

**General experimental procedures:** Optical rotations were measured with an Optical Activity AA-55 polarimeter. UV spectra were measured by a Lengguang Gold S54 photometer. ECD data were collected using JASCO J-715 or J-810 spectropolarimeters. NMR data were recorded on a Bruker Avance 500 MHz spectrometer with TMS as internal standard. Low and high resolution ESI-mass spectra were recorded on a VG Autospec 3000 spectrometer. HPLC analyses were carried out on a Dionex HPLC system (P680 HPLC pump, UVD 340U UV–visible detector) using a C18 column (5 μm, 8.0 mm i.d. × 250 mm). Commercial silica gel (100−200 mesh and 200−300 mesh) for column chromatography were purchased from Qingdao Haiyang Chemical Group Corporation. RP-18 reversed-phase silica gel (40−63 μm) and Sephadex LH-20 were purchased from the Merck Corporation.

**Fungal material:** The isolation and identification of the fungus *P. variotii* EN-291 have been described previously [9].

**Fermentation, extraction and isolation:** The fungus was statically cultivated in a 1000 mL Erlenmeyer flask containing 300 mL of the PDB medium (potato dextrose broth: 2% mannitol, 1% glucose, 0.3% peptone, 0.5% yeast extract, and 300 mL of seawater, 60 flasks) for 30 days at room temperature. The fermented substrate (18 L) was extracted repeatedly with EtOAc (3 × 15 L) to afford a residue (4.3 g), which was subjected to silica gel chromatography using a VLC column with a stepwise gradient of a mixture of petroleum ether (PE)−ethyl acetate (EtOAc) (from 5:1 to 1:1), and then by CHCl_3_−MeOH (20:1 and 10:1) to provide 7 fractions (1−7). Fraction 3 (2.0 g), eluted with petroleum ether−EtOAc (3:1, v/v), was purified by column chromatography (CC) (silica gel, CHCl_3_–MeOH gradient, from 50:1 to 10:1) to obtain five subfractions (3.1−3.5). Fraction 3.3 (200 mg) was further separated by Lobar LiChroprep RP-18 from MeOH–H_2_O 4:6 to 7:3, and finally Sephadex LH-20 (MeOH) to afford compounds **1** (16.0 mg) and **2** (2.2 mg).

**Varioloid A (1):** Light brown solid; [α]_D_^25^ +38 (*c* 0.13, MeOH); UV (MeOH) λ_max_ (log ε): 202 (4.56), 221 (4.57), 289 (4.04) nm; ECD (MeCN, λ [nm] (Δε), *c* = 1.8 mM): 313 (+3.78), 275 (−1.30), 257 (+0.23), 229 (−11.37), 208 (+2.71), 194 (+4.70); ^1^H NMR (500 MHz, CDCl_3_, δ, ppm) and ^13^C NMR (125 MHz, CDCl_3_, δ, ppm) data, see Table 1. HRMS–ESI (*m/z*): [M *+* H]^+^ calcd for C_26_H_24_N_2_O_5_, 445.1758; found, 445.1766.

**Varioloid B (2):** Light brown solid; [α]_D_^25^ +36 (*c* 0.17, EtOH) (lit. [α]_D_^18^ +34.3 (*c* 0.11, EtOH)) [13]; UV (MeOH) λ_max_ (log ε): 202 (4.71), 221 (4.72), 289 (4.17) nm; ECD (MeCN, λ [nm] (Δε), *c* = 0.56 mM): 312 (+5.34), 274 (−1.77), 256 (+0.20), 229 (−11.93), 206 (+4.14), 192 (+3.20); ^1^H NMR (500 MHz, CDCl_3_, δ, ppm) and ^13^C NMR (125 MHz, CDCl_3_, δ, ppm) data, see Table 1.

**Computational methods:** Mixed torsional/low-mode conformational searches were carried out by means of the Macromodel 9.9.223 software [20] using the Merck Molecular Force Field (MMFF) with an implicit solvent model for CHCl_3_ applying a 21 kJ/mol energy window. Geometry reoptimizations of the resultant conformers [B3LYP/6-31G(d) level in vacuo and B97D/TZVP [16–17] with a solvent model (PCM) for MeCN] and TDDFT calculations were performed with Gaussian 09 [21] using various functionals (B3LYP, BH&HLYP, PBE0) and the TZVP basis set. ECD spectra were generated as the sum of Gaussians [22] with 3000 cm^−1^ half-height width (corresponding to ca. 15 nm at 225 nm), using dipole-velocity-computed rotational strengths. Boltzmann distributions were estimated from the ZPVE-corrected B3LYP/6-31G(d) energies in the gas-phase calculations and from the B97D/TZVP energies in the PCM model ones. Torsional energy scans and TS calculations were carried out at the B3LYP/6-31G(d) level in vacuo. The MOLEKEL [23] software package was used for visualization of the results.

**Cytotoxicity assay:** The cytotoxic activities against A549, HCT116, and HepG2 cell lines were determined by the MTT (3-(4,5-dimethylthiazol-2-yl)-2-5-diphenyltetrazolium bromide) assay according to previously reported methods [24–25]. Briefly, the cells cited above were grown in RPMI 1640 (Sigma R6504) medium supplemented with 10% fetal calf serum (Gibco 16000-044) at 37 °C in humidified air with 5% CO_2_. Then the cell lines were treated with test compounds for 48 h, and subsequently MTT solution was added. After incubation for 3 h, the blue formazan generated was solubilized with 0.04 M HCl in isopropanol. The absorbance at 570 nm was read in a Synergy ELISA plate reader (Bio Tek Instruments).

## Supporting Information

File 1 Selected 1D and 2D NMR spectra of compounds **1** and **2**, and computed solvent model ECD spectrum of compound **1**.
