# Correction: Varioloid A, a new indolyl-6,10b-dihydro-5a*H*-[1]benzofuro[2,3-*b*]indole derivative from the marine alga-derived endophytic fungus *Paecilomyces variotii* EN-291

**DOI:** 10.3762/bjoc.14.215

**Published:** 2018-09-12

**Authors:** Peng Zhang, Xiao-Ming Li, Xin-Xin Mao, Attila Mándi, Tibor Kurtán, Bin-Gui Wang

**Affiliations:** 1 Laboratory of Marine Biology and Biotechnology, Qingdao National Laboratory for Marine Science and Technology, Key Laboratory of Experimental Marine Biology, Institute of Oceanology, Chinese Academy of Sciences, Nanhai Road 7, Qingdao 266071, China, Fax: +86 532 82880645; 2 Tobacco Research Institute of Chinese Academy of Agricultural Sciences, Qingdao 266101, China; 3 Department of Organic Chemistry, University of Debrecen, P. O. Box 400, 4002 Debrecen, Hungary, Fax: +36 52 512-744

**Keywords:** bisindolyl benzenoid derivatives, cytotoxicity, marine alga-derived fungus, Paecilomyces variotii, TDDFT-ECD calculation

The authors wish to rename compounds **1** and **2** (shown in Figure 1 of the original article [1]) as varioloids C and D, respectively (Figure 1), as the synonyms varioloids A and B have already been assigned by us to two other compounds in another publication [2]. We thank Andrew Robert from CRC Press for bringing this problem to our attention and apologize for any inconvenience caused.

**Figure 1:**
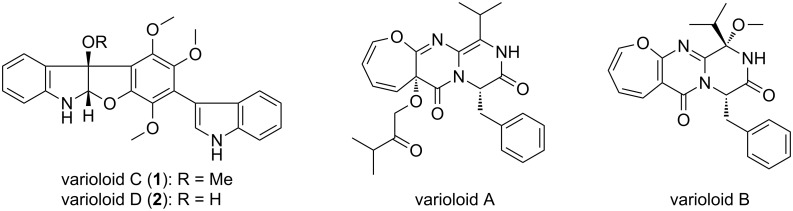
Varioloids A–D.
