# Supplementary material for: Varioloid A, a new indolyl-6,10b-dihydro-5aH-[1]benzofuro[2,3-b]indole derivative from the marine alga-derived endophytic fungus Paecilomyces variotii EN-291
Source: Beilstein J Org Chem. 2016 Sep 9;12:2012–8. doi: 10.3762/bjoc.12.188 (PMC5082447; doi:10.3762/bjoc.12.188)
Supplement: File 1 — Selected 1D and 2D NMR spectra of compounds 1 and 2, and computed solvent model ECD spectrum of compound 1. [file Beilstein_J_Org_Chem-12-2012-s001.pdf]

Supporting Information

for

Varioloid A, a new indolyl-6,10b-dihydro-5a*H*-[1]benzofuro[2,3-*b*]indole derivative from the marine alga-derived endophytic fungus *Paecilomyces variotii* EN-291

Peng Zhang,[1,2] Xiao-Ming Li,[1] Xin-Xin Mao,[2] Attila Mándi,[3] Tibor Kurtán*[3] and Bin-Gui Wang*[1]

Address: [1]Laboratory of Marine Biology and Biotechnology, Qingdao National Laboratory for Marine Science and Technology, Key Laboratory of Experimental Marine Biology, Institute of Oceanology, Chinese Academy of Sciences, Nanhai Road 7, Qingdao 266071, China, Fax: +86 532 82880645, [2]Tobacco Research Institute of Chinese Academy of Agricultural Sciences, Qingdao 266101, China and [3]Department of Organic Chemistry, University of Debrecen, P. O. Box 400, 4002 Debrecen, Hungary, Fax: +36 52 512-744

Email: Tibor Kurtán - kurtan.tibor@science.unideb.hu; Bin-Gui Wang - wangbg@ms.qdio.ac.cn

*Corresponding author

Selected 1D and 2D NMR spectra of compounds 1 and 2, and computed solvent model ECD spectrum of compound 1

Table of contents

Figure S1 HR-ESI-MS spectrum of compound **1**.
Figure S2 ^{1}H NMR (500 MHz, $CDCl_3$) of compound **1**.
Figure S3 ^{13}C NMR and DEPT (125 MHz, $CDCl_3$) of compound **1**.
Figure S4 COSY spectrum of compound **1**.
Figure S5 HSQC spectrum of compound **1**.
Figure S6 HMBC spectrum of compound **1**.
Figure S7 NOESY spectrum of compound **1**.
Figure S8 Computed solvent model ECD spectrum of compound **1**.
Figure S9 ^{1}H NMR (500 MHz, $CDCl_3$) of compound **2**.
Figure S10 ^{1}H NMR (500 MHz, DMSO-d_6) of compound **2**.
Figure S11 ^{13}C NMR and DEPT (125 MHz, $CDCl_3$) of compound **2**.
Figure S12 COSY spectrum of compound **2**.
Figure S13 HSQC spectrum of compound **2**.
Figure S14 HMBC spectrum of compound **2**.
Figure S15 NOESY spectrum (DMSO-d_6) of compound **2**.

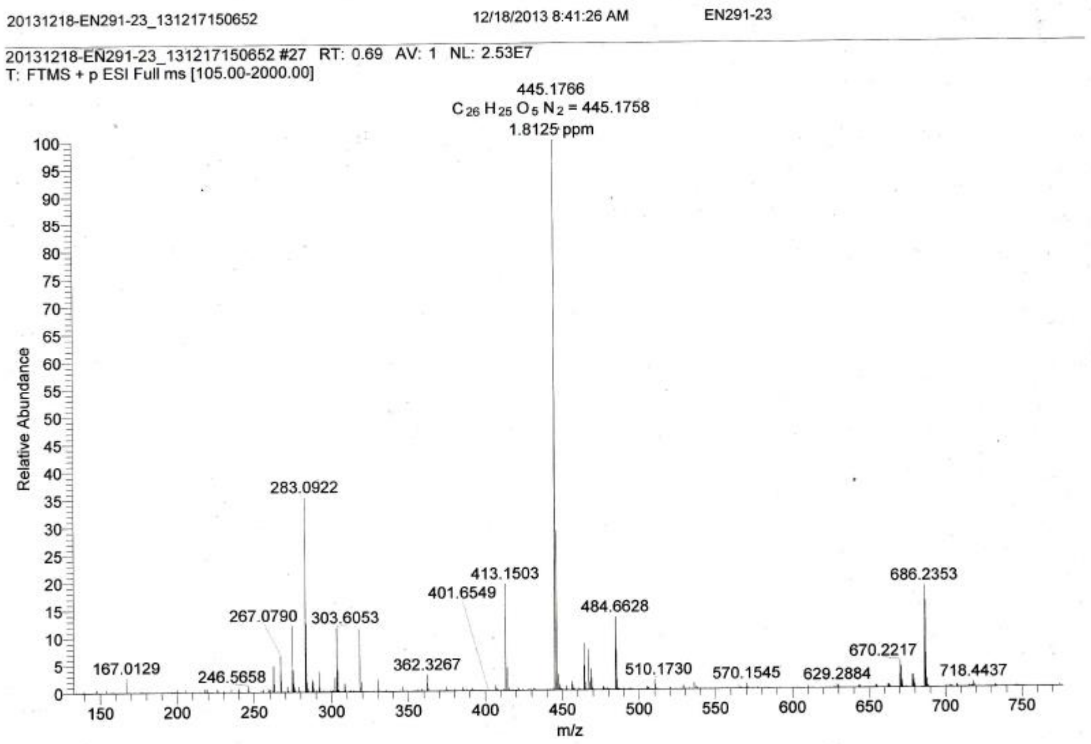

Figure S1: HRMS–ESI spectrum of compound **1**.

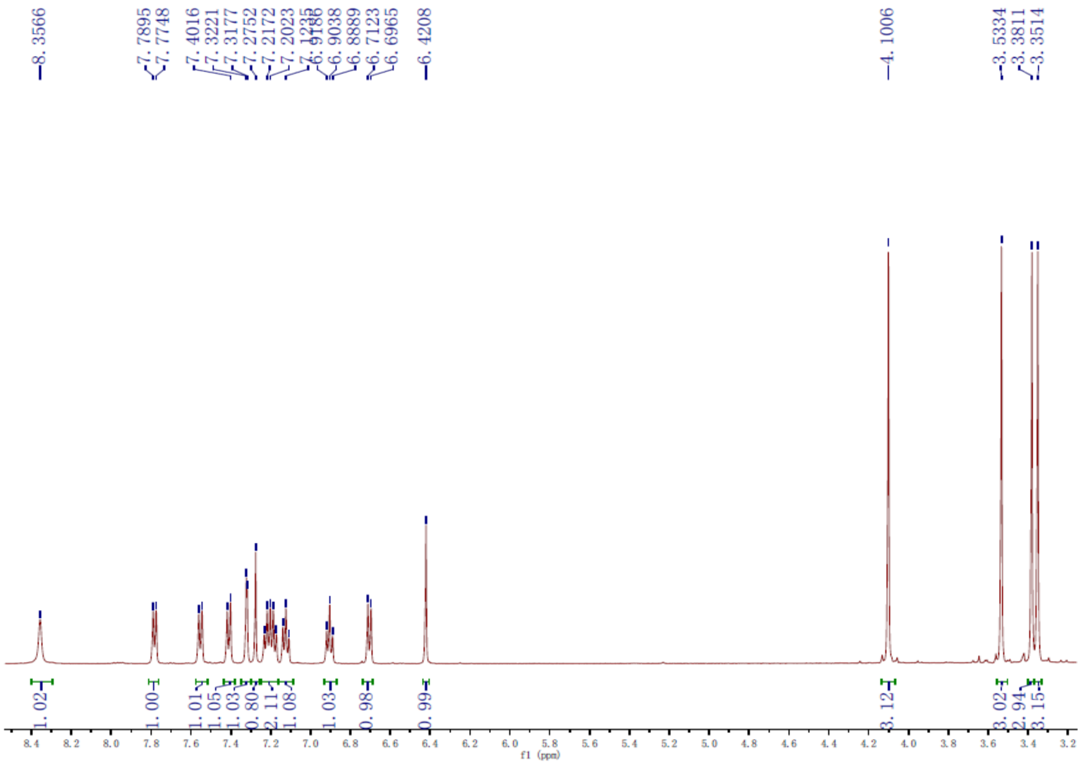

Figure S2: ^{1}H NMR (500 MHz, $CDCl_3$) of compound **1**.

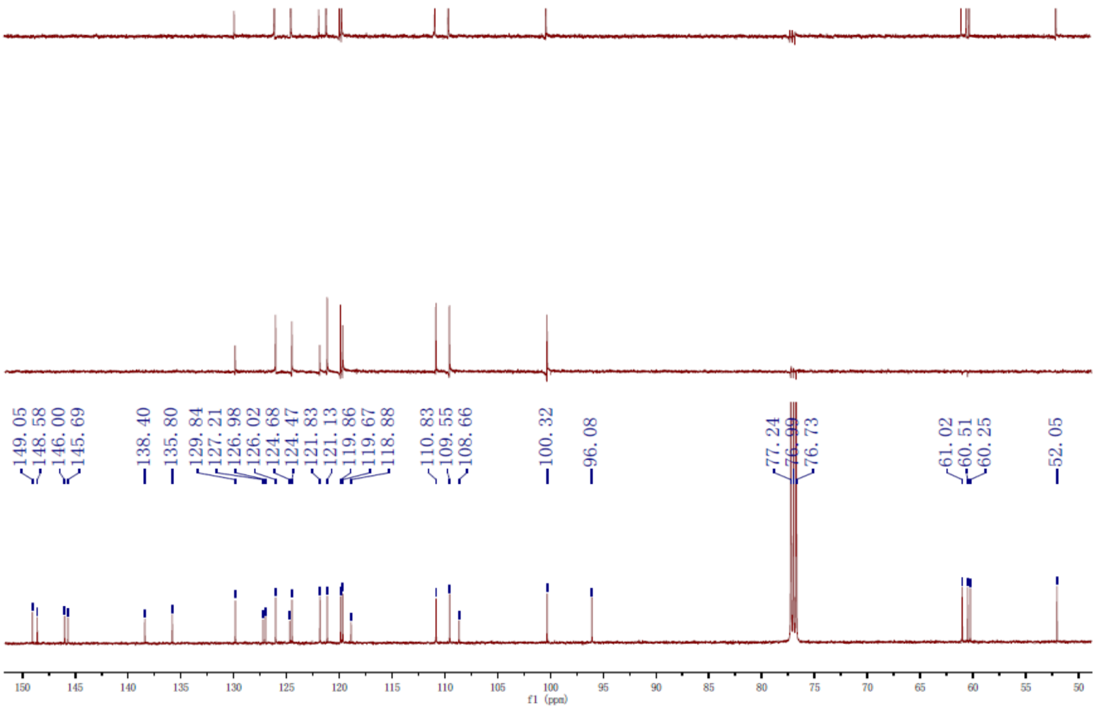

Figure S3: ^{13}C NMR and DEPT (125 MHz, $CDCl_3$) of compound **1**.

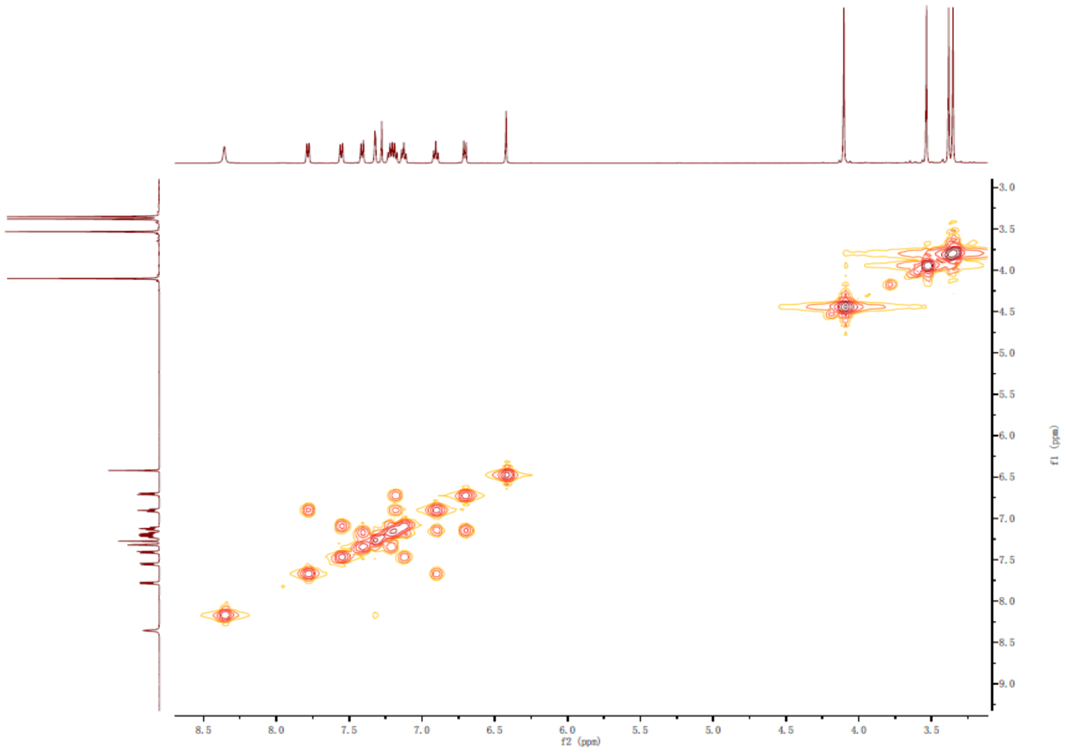

Figure S4: COSY spectrum of compound **1**.

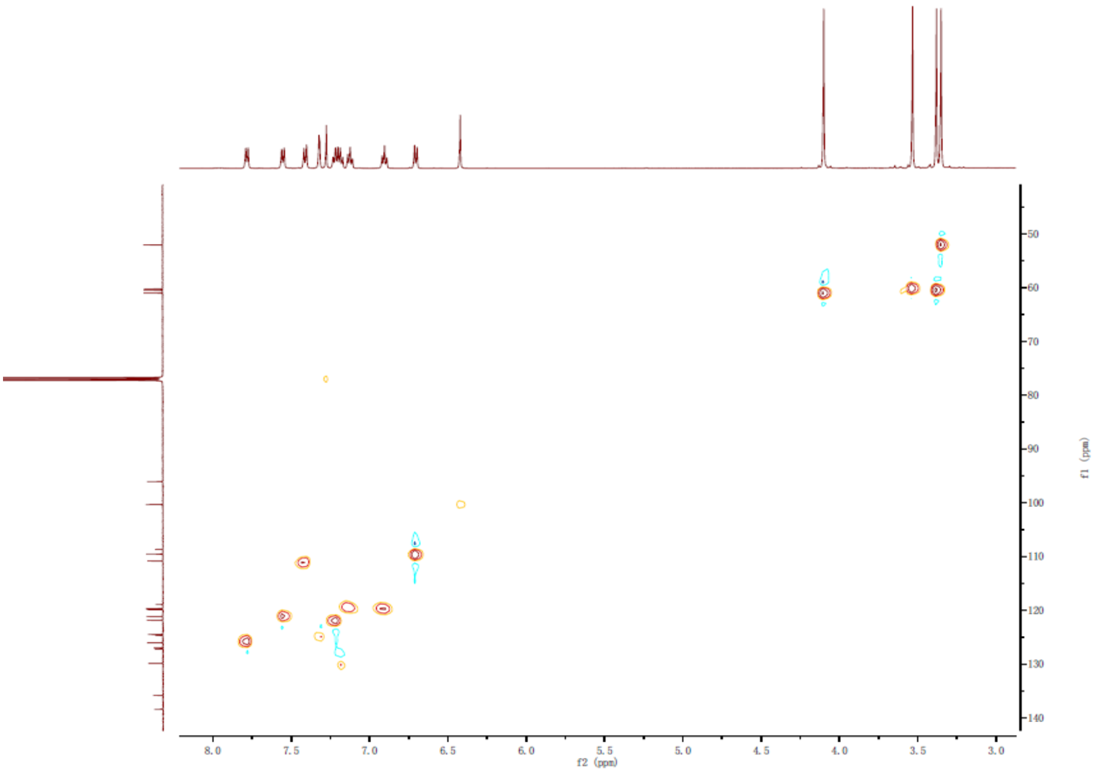

Figure S5: HSQC spectrum of compound **1**.

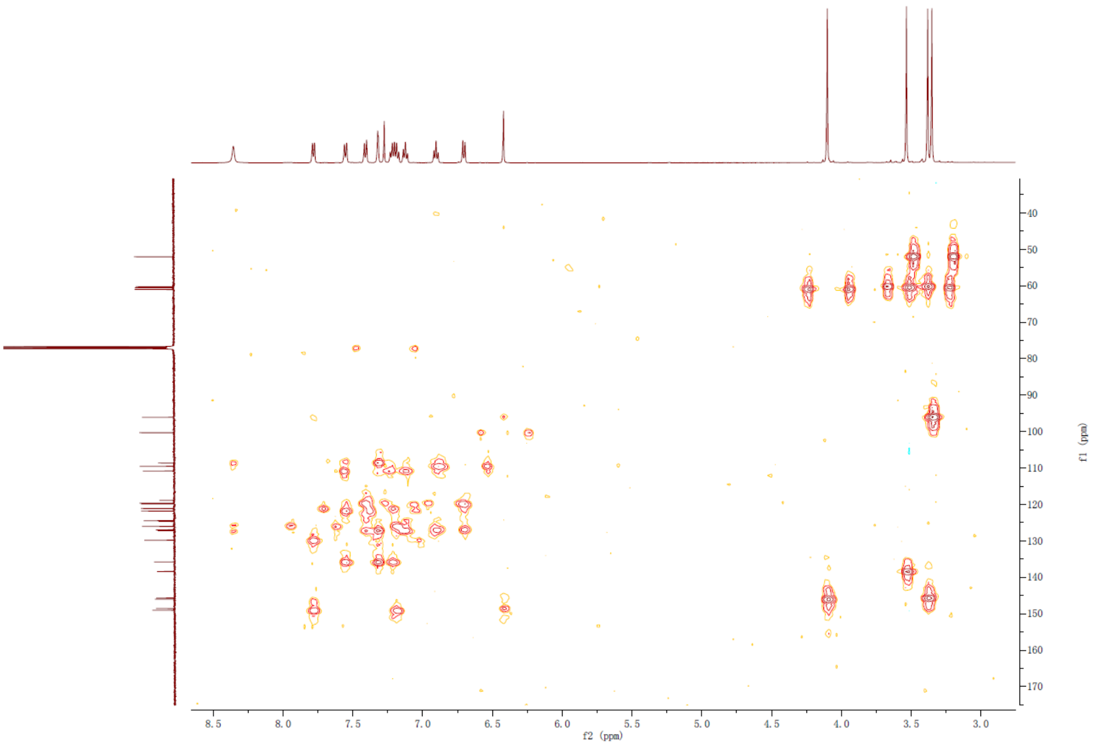

Figure S6: HMBC spectrum of compound **1**.

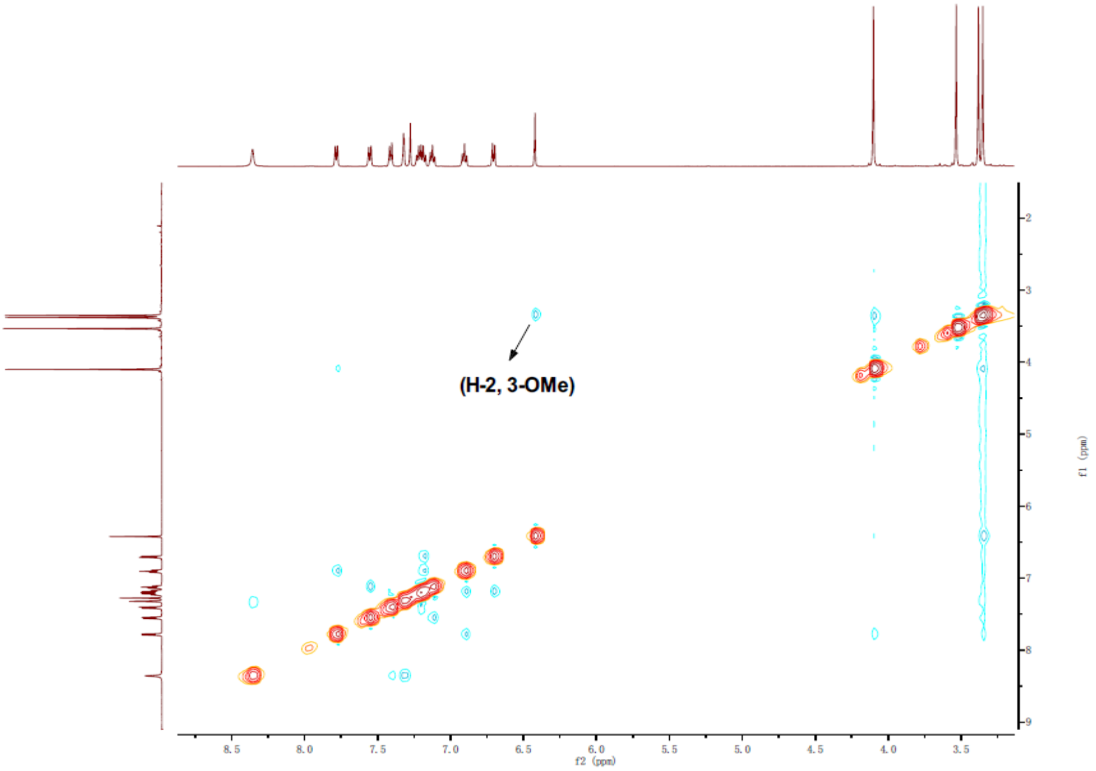

Figure S7: NOESY spectrum of compound **1**.

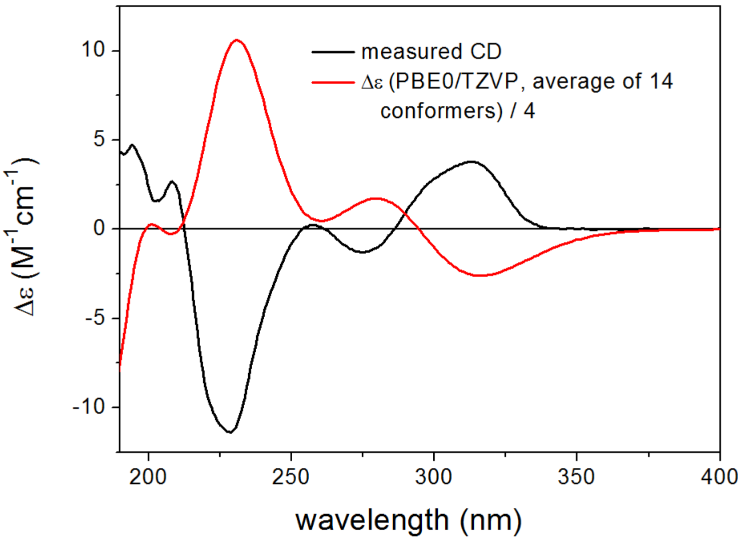

Figure S8: Computed solvent model ECD spectrum (PBE0/TZVP PCM/MeCN) of compound **1**. Level of DFT optimization: B97D/TZVP PCM/MeCN.

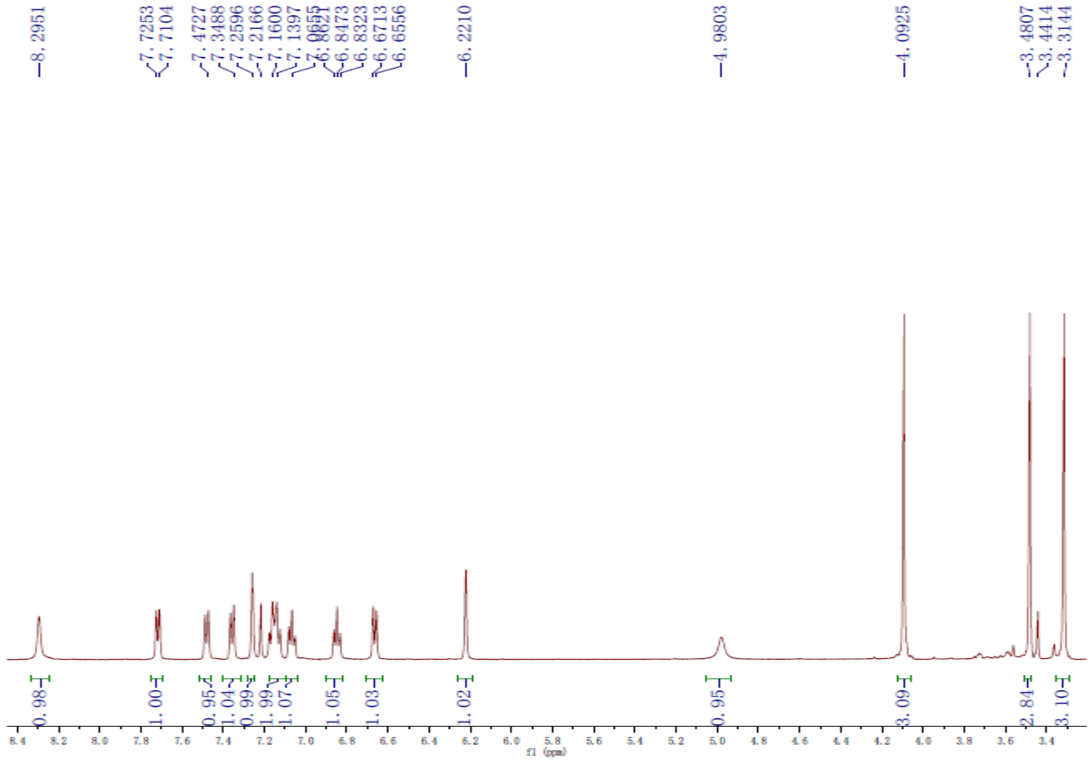

Figure S9: ^{1}H NMR (500 MHz, $CDCl_3$) of compound **2**.

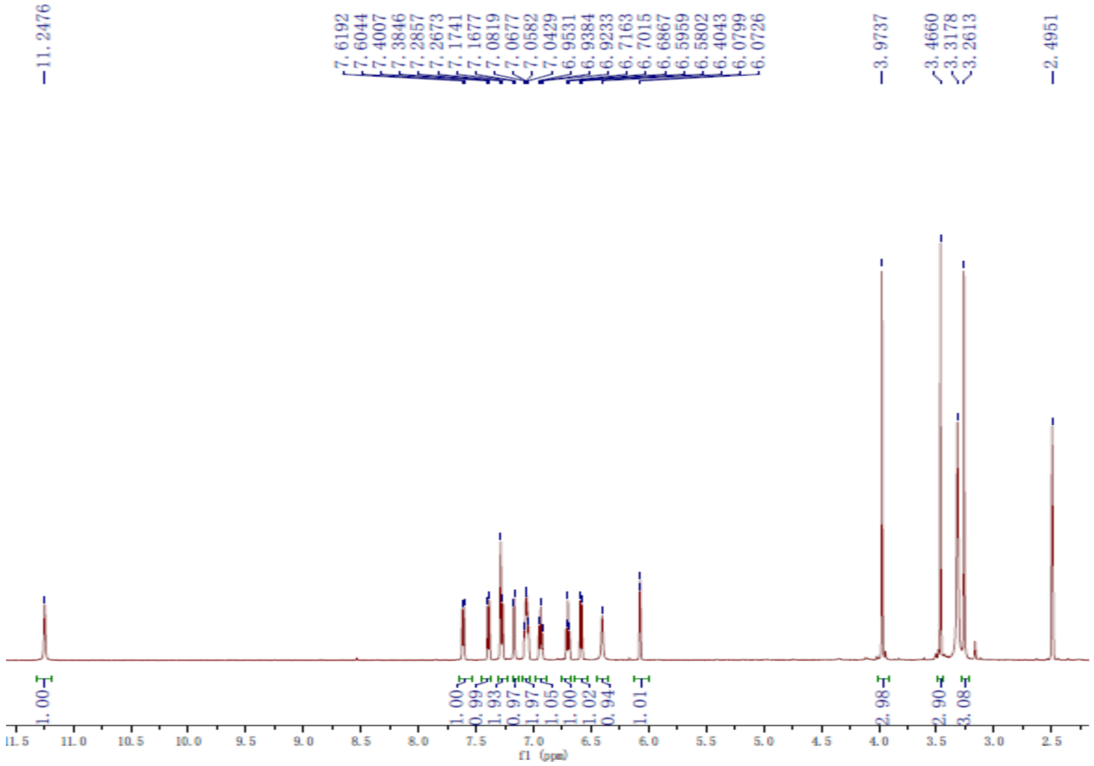

Figure S10: ^{1}H NMR (500 MHz, DMSO-d_6) of compound **2**.

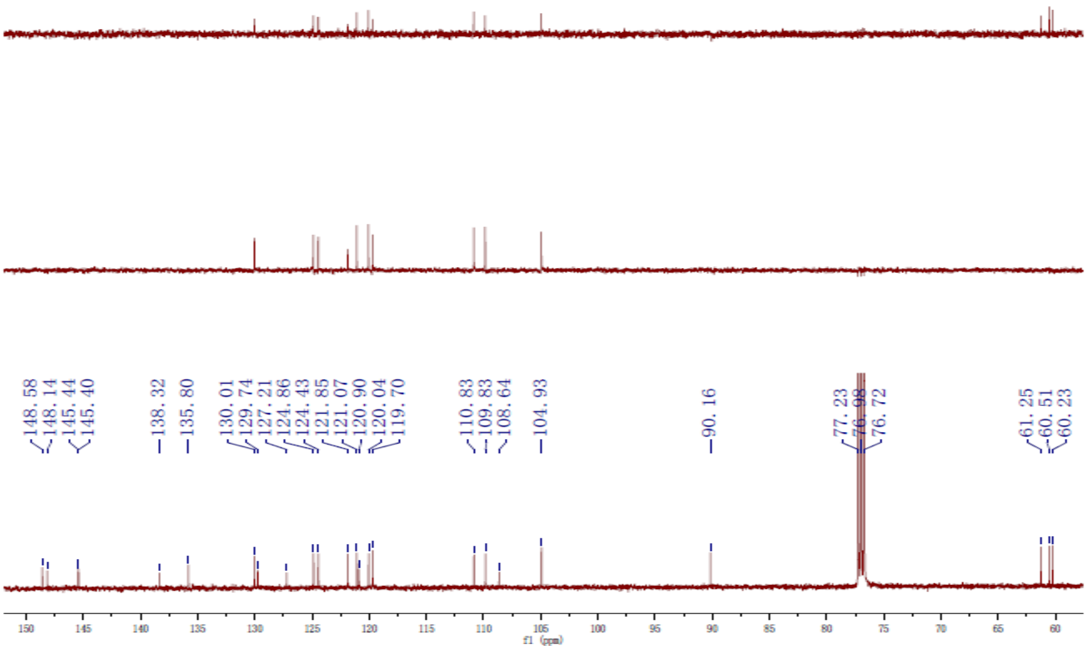

Figure S11: ^{13}C NMR and DEPT (125 MHz, $CDCl_3$) of compound **2**.

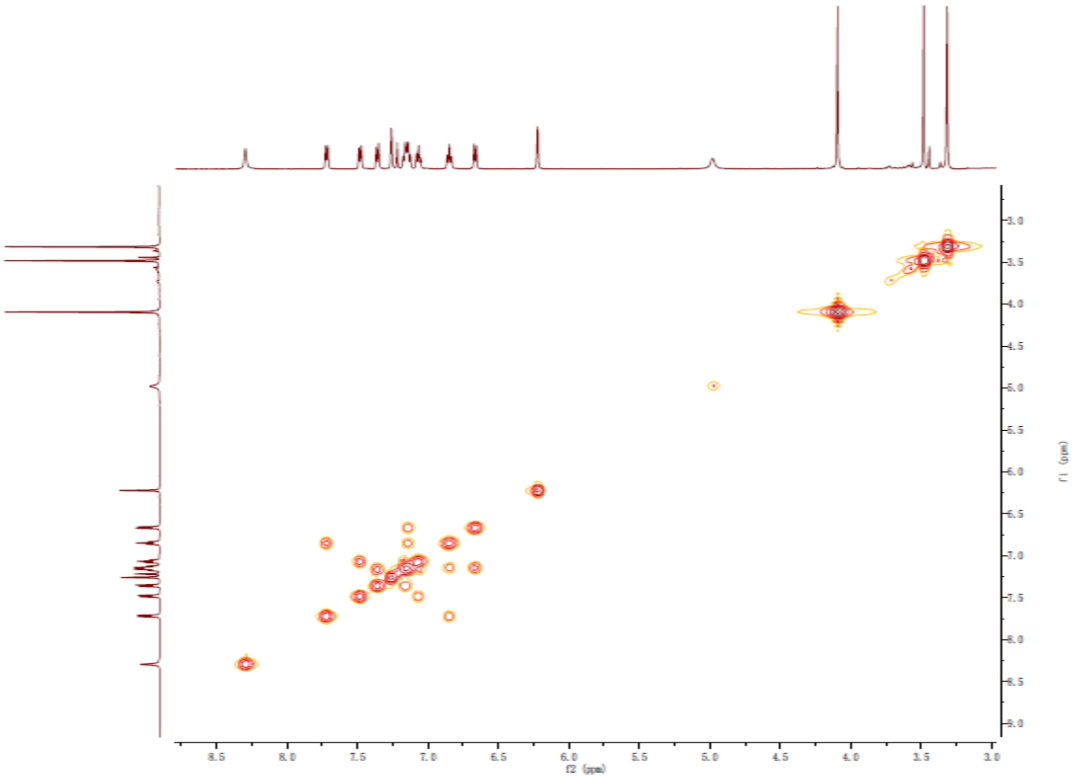

Figure S12: COSY spectrum of compound **2**.

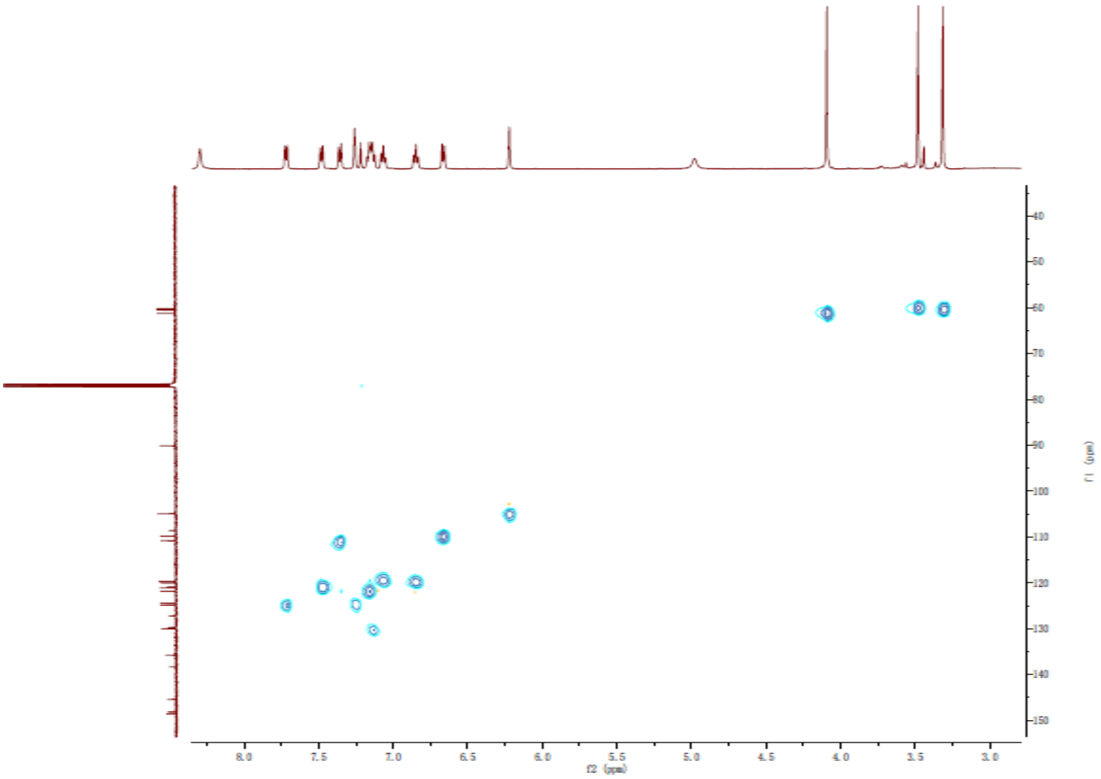

Figure S13: HSQC spectrum of compound **2**.

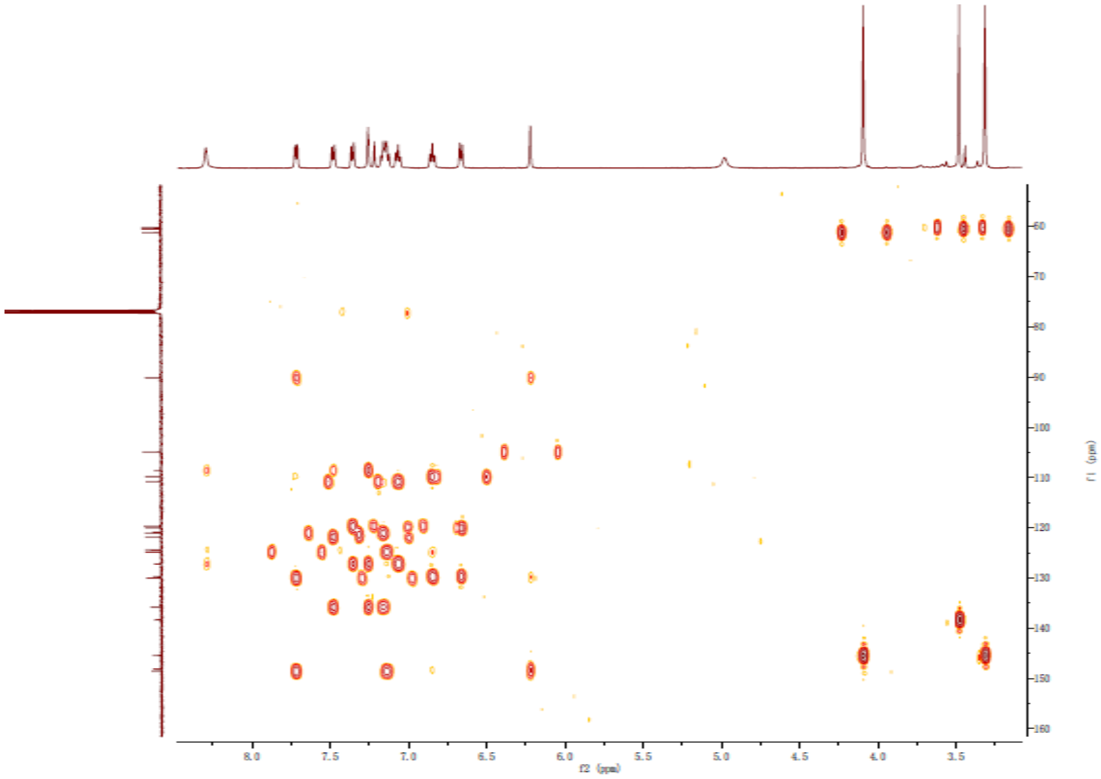

Figure S14: HMBC spectrum of compound **2**.

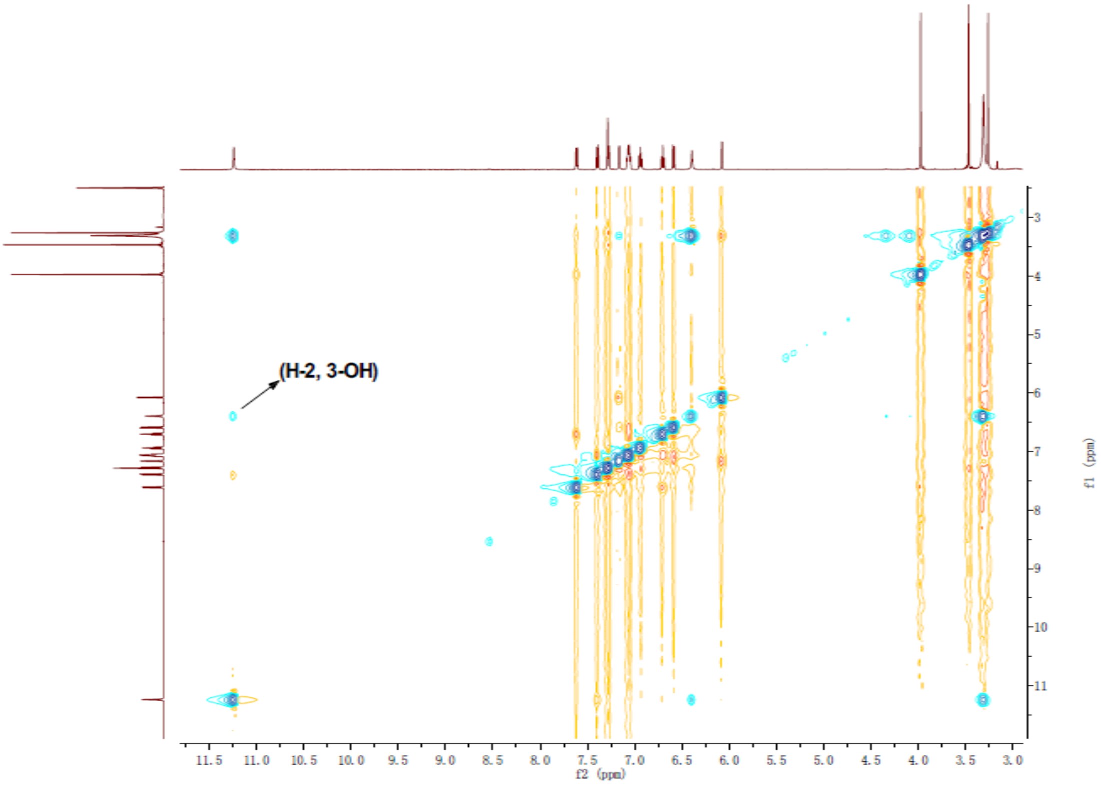

Figure S15: NOESY spectrum (DMSO-d_6) of compound **2**.
